# Validation of plasma D-dimer in Chinese patients with acute non-ST segment elevation myocardial infarction

**DOI:** 10.3389/fcvm.2022.896173

**Published:** 2022-10-19

**Authors:** Xin Fan, Tingting Min, Shaohui Su, Bin Xiong, Huaibin Wan

**Affiliations:** ^1^Department of Cardiology, Dongguan People's Hospital, The First School of Clinical Medicine, Southern Medical University, Dongguan, China; ^2^Department of Cardiology, Heyuan Shenhe People's Hospital, The Fifth Affiliated Hospital of Jinan University, Guangzhou, China

**Keywords:** D-dimers, acute non-ST-segment myocardial infarction, coronary artery diseases, outcomes, retrospective study

## Abstract

**Objective:**

To analyze the predictive values of D-dimer in Chinese patients with non-ST-segment elevation myocardial infarction (NSTEMI).

**Methods:**

We retrospectively retrieved consecutive patients hospitalized due to acute NSTEMI from January 2015 to December 2018 from the Electronic Medical Record (EMR) library. Clinical and follow-up data were collected. The primary endpoint was major adverse composite cardiovascular events (MACEs), such as all-cause death, non-fatal myocardial infarction, and non-fatal stroke. The secondary endpoints included all-cause death, non-fatal myocardial infarction, non-fatal stroke, heart failure, and severe arrhythmias. The Cox regression model was used to evaluate the association between risk factors and clinical outcomes in Chinese patients with NSTEMI.

**Results:**

A total of 673 patients were included; the median age was 64.0 (53.0–75.0) years old and 76.2% were men. Patients with higher D-dimer levels were more often women, older, had a higher Charlson Comorbidity Index, and had a higher incidence of MACEs (59.9 vs. control 9.0%; *p* < 0.001) and all-cause death (49.1 vs. control 2.2%; *p* < 0.001). The multivariate Cox analysis suggested that the D-dimer level was an independent predictor of MACEs (hazard ratio [HR]: 1.069, 95% CI: 1.010–1.132, *p* = 0.021). The receiver operating characteristic (ROC) analysis suggested that D-dimer levels were better than the Charlson Comorbidity Index in all-cause death.

**Conclusion:**

In Chinese patients with acute NSTEMI, higher D-dimer levels on admission suggest a poor long-term prognosis.

## Introduction

Acute myocardial infarction is a common critical illness based on atherosclerotic thrombosis and is a leading cause of morbidity and mortality (1). Patients exhibit increased coagulation system activity during the acute phase of unstable angina and myocardial infarction and manifest a persistent hypercoagulable state with minimal generation of fibrin over the next 6 months (2). Plasma D-dimer, a soluble degradation product of cross-linked fibrin during fibrinolysis (3), is a specific marker of thrombosis and indicates a hypercoagulable state. Previous studies reported that D-dimer correlates with vascular endothelial dysfunction and is a risk factor for the onset of major adverse composite cardiovascular events (MACEs) and all-cause death (4, 5). Several studies have suggested that increased D-dimer levels are associated with the presence and severity of coronary disease (6–8) and indicate an unfavorable prognosis for stable coronary disease (9). Therefore, D-dimer may be a valuable marker for predicting ischemic events (10). However, to our knowledge, the role of D-dimer in predicting the incidence of adverse events in Chinese patients with non-ST-segment elevation myocardial infarction (NSTEMI) has not been thoroughly studied. Therefore, we explored the predictive value of D-dimer on the prognosis of Chinese patients with NSTEMI based on our previous clinical data.

## Methods

### Study population

This was a retrospective study designed to investigate associated risk factors and clinical outcomes among Chinese patients with NSTEMI. We retrospectively retrieved and anonymously obtained the consecutive hospitalized patients due to acute NSTEMI between January 2015 and December 2018 from the Electronic Medical Record (EMR) library of Dongguan People's Hospital. The inclusion criteria were as follows: (1) age >18 years and (2) a diagnosis of NSTEMI based on the International Classification of Diseases 11th Revision (ICD-11) (such as, BA41, BA41.1, and BA41); (3) meets the fourth universal definition of myocardial infarction (11); and (4) the onset of myocardial infarction was less than 48 h. Exclusion criteria were as follows: (1) an electrocardiogram (ECG) with persistent ST-segment elevation in at least two consecutive leads; (2) duplicate cases; and (3) patients who were lost during the first 1-year follow-up period. This study is exempt from the need for informed consent according to China's “Ethical Review Approaches for Biomedical Research Involving Humans” 2016, Article 39 (1) (12). The study was approved by the institute ethics committee and was conducted following the Declaration of Helsinki (Ethics Approval Number: KYKT2022-012).

### Data collection and laboratory measurements

The baseline clinical characteristics included the patient's gender, age, smoking history, systolic blood pressure (SBP), diastolic blood pressure (DBP), blood glucose (BG), body mass index (BMI = weight (kg)/ [height (m)]^2^), and past medical histories. The age-adjusted Charlson Comorbidity Index (ACCI) (13, 14) was introduced to weigh the burden of comorbidities. Blood samples were drawn from fasting patients by venipuncture within 24 h after admission and stored in 3.2% citrated tubes or plain tubes according to the clinical laboratory requirements. Plasma D-dimer and fibrinogen (Fib) were detected by latex agglutination turbidimetry using a STARMAX automatic coagulation apparatus (STAGO, France). The normal range of plasma D-dimer levels was < 0.5 μg/ml. Low-density lipoprotein cholesterol (LDL-C) was measured using clinical laboratory methods (Beckman CX9, USA). C-reactive protein (CRP) was measured based on an immunofluorescence dry quantitative method using an automatic dry immunoassay analyzer (A5000, Boditech Biotechnology, China). B-Type natriuretic peptide (BNP) was measured by UniCel Dxi800 Access (Beckman Coulter, China). Troponin T (TNT) was measured using a Roche Cobas h 232 immunoassay analyzer (Roche Diagnostics, Mannheim, Germany). The left ventricular ejection fraction (LVEF) was estimated using the Simpson method.

### Follow-up and outcomes

A clinical follow-up visit was performed every 3 months by the treating physicians. An independent research team evaluated patients' status and end-point events according to the follow-up records. Any inconsistencies were resolved by the researcher through telephone interviews with the treating physician and/or patients. The follow-up period ended at death or November 2021. The primary endpoint event was defined as a major adverse cardiovascular event (MACE), which included non-fatal myocardial infarction, non-fatal cerebral infarction, and all-cause death. The secondary endpoint events were single events, such as all-cause death, non-fatal myocardial infarction, non-fatal stroke, heart failure, and severe arrhythmias.

### Statistical analysis

Patients were grouped according to their quartiles of plasma D-dimer level at admission. The normal distribution of continuous variables was verified by the Kolmogorov–Smirnov test. Continuous variables are presented as the mean ± standard deviation (SD) or median and interquartile range (IQR) and were tested by one-way analysis of variance (ANOVA) or the Mann–Whitney *U*-test, as appropriate. The count data are presented as frequencies (*n*) and percentages (%), and the comparisons of the groups were conducted using the χ^2^ test. Patient survival in different groups was described by Kaplan–Meier analyses and log-rank tests. Risk factors for endpoint events were assessed using a univariate and/or multivariate Cox regression analysis. A *p-*value < 0.05 was considered statistically significant (a two-tailed test). Analyses were performed using the SPSS 22.0 software package for Windows (IBM, USA).

## Results

**Figure 1:**
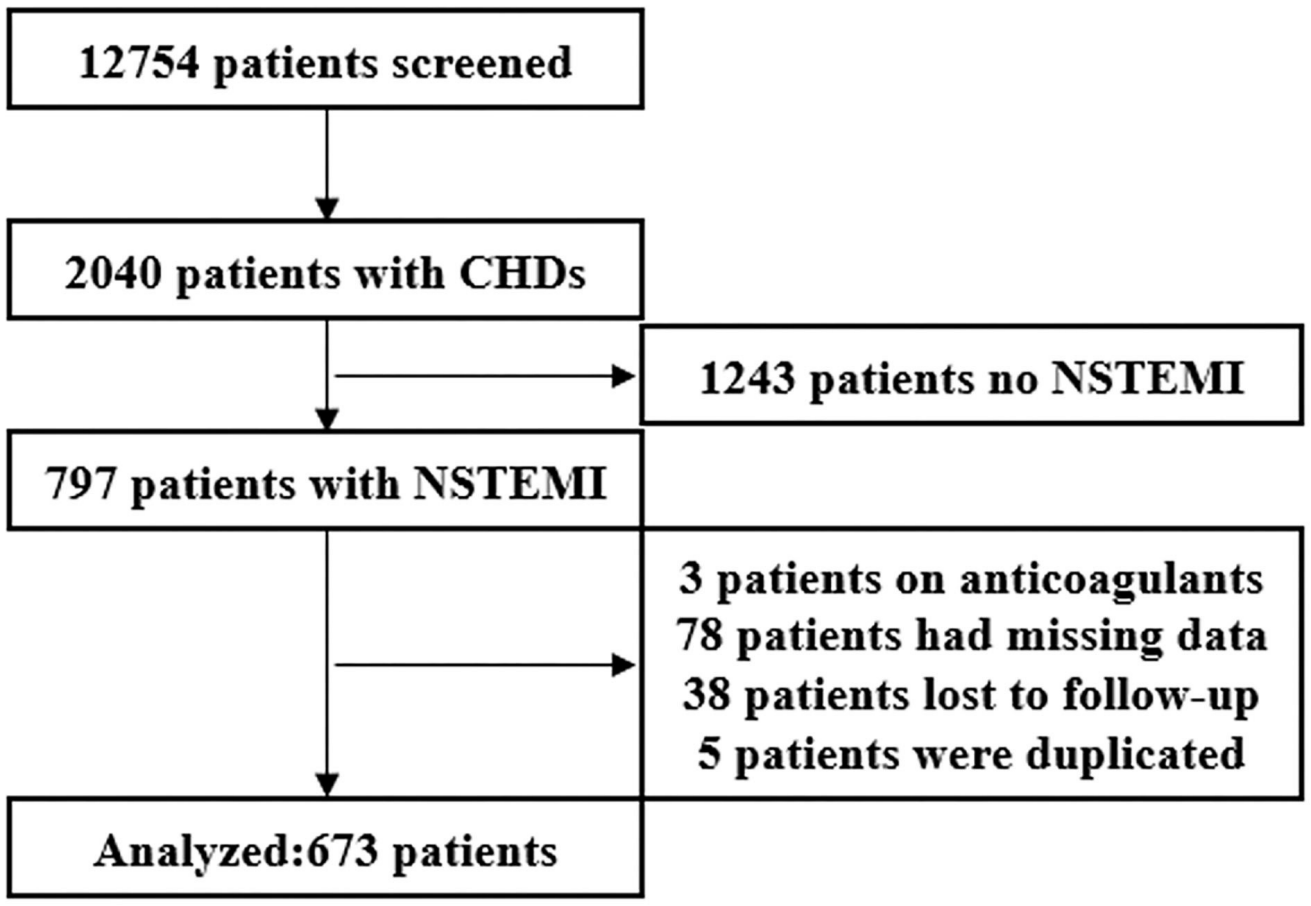
A flow diagram of the study.

According to the inclusion and exclusion criteria, a total of 673 patients (Figure 1) were finally included (median age, 64.0 (53.0–75.0) years; men, 76.2%; and BMI, 24.62 ± 3.40 kg/m^2^). The baseline median plasma D-dimer level was 0.46 (0.30–0.96) mg/dl. A total of 60.2% (405/673) of patients had a history of smoking, 62.6% (421/673) of patients had a history of hypertension, and 33.4% (225/673) of patients had a history of diabetes.

The median DBP was 72.00 (83.00–96.00) mmHg. The median LVEF was 62.00 (55.00–68.00)%. The median fibrinogen was 3.75 (3.16–4.74) g/L. The median CRP level was 145 (62.50–427.90). The median ACCI was 4 (3–6). The baseline characteristics of the patients are listed in Table 1. Table 2 shows the medication status of the four groups. Except for antihyperlipidemic agents, there were significant differences in the use of the other 5 drugs among the groups.

**Table 1:** Baseline characteristics of patients with non-ST-segment elevation myocardial infarction (NSTEMI).

**Variable**	**Total (*n* = 673)**	**D-dimer quartile**				***P*-value**
		**Q1 (*n* = 178)**	**Q2 (*n* = 167)**	**Q3 (*n* = 161)**	**Q4 (*n* = 167)**	
D-dimer,μg/ml	0.46 (0.30–0.96)	0.23 (0.22–0.27)	0.37 (0.34–0.41)	0.65 (0.54–0.79)	1.88 (1.29–2.78)	< 0.001
Men, *n* (%)	513 (76.2)	155 (87.1)	136 (81.4)	118 (73.3)	104 (62.3)	< 0.001
Age, years	64.00 (53.00–75.00)	54.00 (46.00–62.00)	59.00 (51.00–66.00)	68.5 (57.00–77.25)	76.00 (68.25–84.00)	< 0.001
BMI, kg/m^2^	24.62 ± 3.40	24.99 ± 3.22	24.85 ± 3.29	24.37 ± 3.15	24.09 ± 3.96	0.112
SBP, mmHg	141.00 (122.00–161.00)	140.50 (122.00–160.75)	144.00 (130.00–161.00)	147.00 (130.00–170.00)	145.00 (123.25–168.50)	0.109
DBP, mmHg	72.00 (83.00–96.00)	85.00 (75.00–101.50)	83.00 (77.00–98.00)	83.00 (74.00–95.00)	82.50 (69.50–93.75)	0.002
Smoking, *n* (%)	405 (60.2)	120 (67.4)	105 (62.9)	97 (60.2)	83 (49.7)	0.008
Hypertension, *n* (%)	421 (62.6)	86 (48.3)	106 (63.5)	105 (65.2)	124 (74.3)	< 0.001
Diabetes, *n* (%)	225 (33.4)	49 (27.5)	63 (37.7)	51 (31.7)	62 (37.1)	0.145
Blood glucose, mmol/L	6.30 (5.40–8.21)	6.02 (5.28–7.65)	6.09 (5.32–7.20)	6.50 (5.10–8.10)	6.44 (5.61–9.13)	0.014
LVEF, %	62.00 (55.00–68.00)	64 (60.00–69.00)	62.00 (55.00–67.00)	65.00 (58.00–71.00)	56.50 (46.00–65.00)	< 0.001
LDL-C, mmol/L	3.45 ±1.09	3.50 ±0.99	3.70 ± 1.05	3.41 ± 1.25	3.17 ± 1.08	0.059
Fibrinogen, g/L	3.75 (3.16–4.74)	3.21 (2.88–3.75)	3.74 (3.02–4.24)	4.41 (3.57–5.35)	4.71 (3.58–5.61)	< 0.001
BNP, pg/mL	322.50 (112.00–800.25)	355.50 (150.75–976.25)	364.00 (133.00–894.00)	240.00 (34.78–1057.00)	419.00 (188.75–955.25)	0.403
CRP, mg/L	145.00 (62.50–427.90)	138.75 (64.25–432.45)	193.30 (71.00–342.00)	130.00 (60.90–328.00)	228.50 (79.50–664.75)	0.010
TnT, ng/L	5.27 (3.00–24.40)	3.00 (3.00–20.76)	3.00 (3.00–14.37)	5.85 (3.00–46.32)	6.37 (3.00–35.58)	0.056
ACCI	4 (3–6)	3 (2–4)	3 (2–5)	6 (4–7)	7 (5–8)	< 0.001
PCI, *n* (%)	378 (56.2)	103 (57.9)	92 (55.1)	88 (54.7)	95 (56.9)	0.925

D-dimer quartile: Q1: ≤ 0.3 μg/L; Q2: 0.31–0.46 μg/L; Q3: 0.47–0.96 μg/L; and Q4: >0.96 μg/L.

*BMI*, body mass index; *LDL*-C, low-density lipoprotein cholesterol; *AC*CI, age-adjusted Charlson Comorbidity Index; *BNP*, B-type natriuretic peptide; *CRP*, C-reactive protein; *TnT*, troponin T; *SBP*, systolic blood pressure; *DBP*, diastolic blood pressure; HR, heart rate; *LVEF*, left ventricular ejection fraction; *P*CI, percutaneous coronary intervention.

**Table 2:** Medication status of patients with NSTEMI.

**Drugs**	**Total (*n* = 673)**	**D-dimer quartiles**				***P*-value**
		**Q1 (*n* = 178)**	**Q2 (*n* = 167)**	**Q3 (*n* = 161)**	**Q4 (*n* =167)**	
Aspirin	91.1% (613/673)	93.3% (166/178)	94.0% (157/167)	93.2% (150/161)	83.8% (140/167)	0.002
Clopidogrel	94.2% (634/673)	97.2% (173/178)	97.6% (163/167)	95.0% (153/161)	86.8% (145/167)	< 0.001
Antihyperlipidemic agents	97.0% (653/673)	97.8% (174/178)	97.6% (163/167)	98.8% (159/161)	94.0% (157/167)	0.059
ACEI/ARB	64.2% (432/673)	79.8% (142/178)	69.5% (116/167)	65.8% (106/161)	40.7% (68/167)	< 0.001
β-Blocker	72.5% (488/673)	82.6% (147/178)	80.8% (135/167)	75.8% (122/161)	50.3% (84/167)	< 0.001
CCB	21.5% (145/673)	14.0% (25/178)	18.6% (31/167)	21.1% (34/161)	32.9% (55/167)	< 0.001

D-dimer quartile: Q1: ≤ 0.3 μg/L; Q2: 0.31–0.46 μg/L; Q3: 0.47–0.96 μg/L; and Q4: >0.96 μg/L.

*ACEI*, angiotensin-converting enzyme inhibitors; *ARB*, angiotensin receptor blockers; *CCB*, calcium channel blockers.

**Figure 2:**
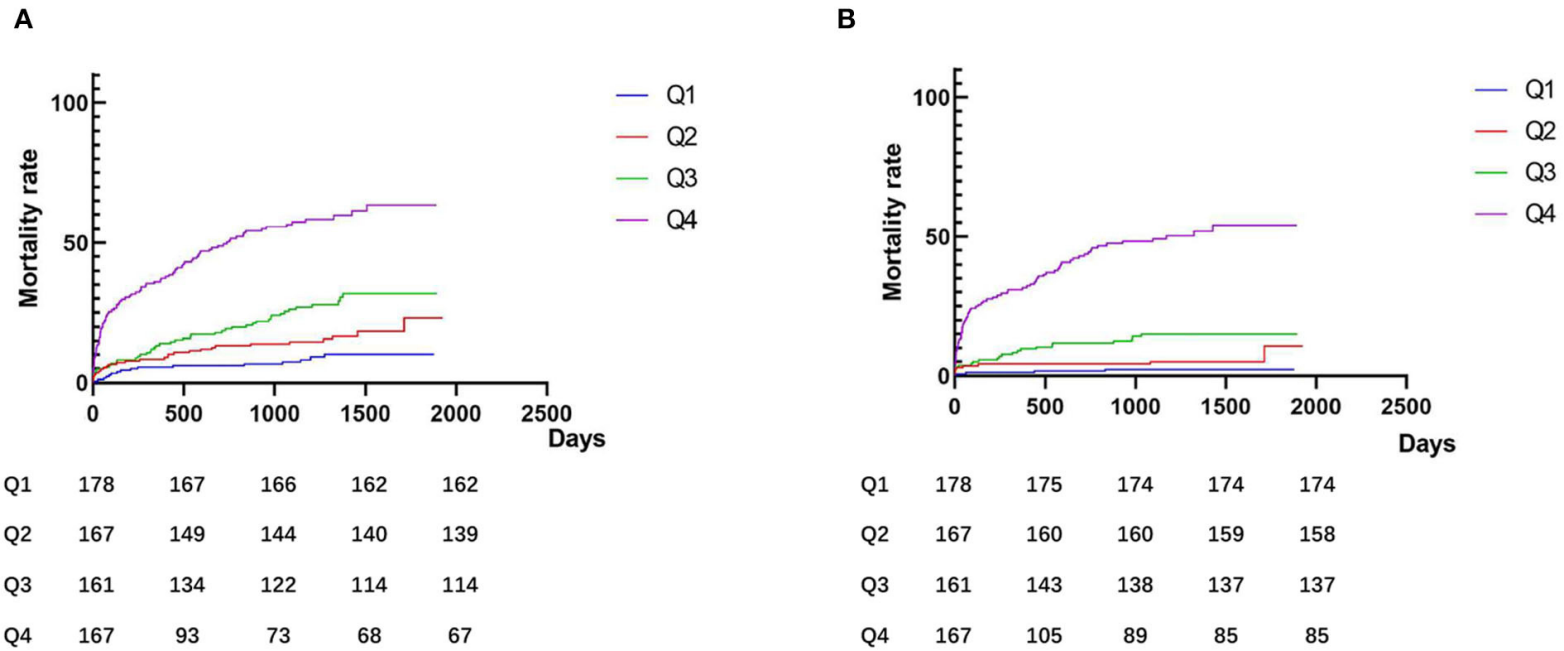
Kaplan–Meier curves according to groups of admission D-dimer levels. **(A)** Major adverse composite cardiovascular events (MACEs); and **(B)** All-cause death. Q1: ≤ 0.3 μg/L; Q2: 0.31–0.46 μg/L; Q3: 0.47–0.96 μg/L; and Q4: >0.96 μg/L.

Patients were followed up for a total of 1,936.4 person·years, and the average follow-up time was 1050.19 ± 548.26 days. During the follow-up period, 191 (28.4%) patients experienced MACEs, 1,119 (17.7%) patients died, 49 (7.3%) patients developed non-fatal myocardial infarction, 20 (3.0%) patients developed non-fatal cerebral infarction, 40 (5.9%) patients suffered heart failure, and 15 (2.2%) patients experienced severe arrhythmias. The incidence of clinical outcomes is shown in Table 3. As shown in the Kaplan–Meier analysis (Figure 2), plasma D-dimer levels were significantly correlated with MACEs (log-rank χ^2^ = 142.9, *p* < 0.001) and all-cause death (log-rank χ^2^ = 181.1, *p* < 0.001).

**Table 3:** Clinical outcomes during the follow-up period.

**Events**	**Total (*n* = 673)**	**D-dimer quartiles**				***P*-value**
		**Q1 (*n* =178)**	**Q2 (*n* =167)**	**Q3 (*n* =161)**	**Q4 (*n* =167)**	
MACEs	28.4% (191/673)	9.0% (16/178)	16.8% (28/167)	29.2% (47/161)	59.9% (100/167)	< 0.001
All-cause death	17.7% (119/673)	2.2% (4/178)	5.4% (9/167)	14.9% (24/161)	49.1% (82/167)	< 0.001
Nonfatal myocardial infarction	7.3% (49/673)	5.1% (9/178)	8.4% (14/167)	8.7% (14/161)	7.2% (12/167)	0.555
Nonfatal cerebral infarction	3.0% (20/673)	1.7% (3/178)	3.0% (5/167)	5.0% (8/161)	2.4% (4/167)	0.351
Heart failure	5.9% (40/673)	3.9% (7/178)	4.2% (7/167)	5.6% (9/161)	10.2% (17/167)	0.055
Severe arrhythmias	2.2% (15/673)	1.1% (2/178)	1.8% (3/167)	2.5% (4/161)	3.6% (6/167)	0.454

Major adverse composite cardiovascular events (MACEs) include all-cause death, non-fatal myocardial infarction, and non-fatal cerebral infarction. D-dimer quartile: Q1: ≤ 0.3 μg/L; Q2: 0.31–0.46 μg/L; Q3: 0.47–0.96 μg/L; and Q4: >0.96 μg/L.

The results of the univariate and multivariate Cox regression analyses of MACEs are listed in Table 4.

**Table 4:** The univariate and multivariate Cox regression analyses of MACEs in patients with NSTEMI.

**Variables**	**Univariable**		**Model 1** ^**#**^		Model 2*	
	**HR (95% CI)**	***P*-value**	**HR (95% CI)**	***P*-value**	**HR (95% CI)**	***P*-value**
Gender	1.487 (1.090–2.027)	0.012	0.955 (0.656–1.390)	0.811	0.948 (0.653–1.378)	0.781
Age, years	1.058 (1.046–1.070)	< 0.001	1.018 (0.998–1.038)	0.081	1.015 (0.995–1.036)	0.150
D-dimer	1.152 (1.120–1.185)	< 0.001	1.069 (1.010–1.132)	0.021	–	
Q2 vs. Q1	1.921 (1.039–3.550)	0.037	–		1.348 (0.649–2.799)	0.423
Q3 vs. Q1	3.561 (2.019–6.281)	< 0.001	–		1.551 (0.770–3.124)	0.220
Q4 vs. Q1	9.998 (5.891–16.969)	< 0.001	–		2.336 (1.144–4.768)	0.020
Smoking	0.869 (0.653–1.157)	0.337				
Fibrinogen	1.272 (1.154–1.402)	< 0.001	1.054 (0.926–1.199)	0.429	0.992 (0.868–1.133)	0.901
ACCI	1.419 (1.339–1.503)	< 0.001	1.262 (1.139–1.399)	< 0.001	1.225 (1.103–1.360)	< 0.001
BG	1.004 (0.994–1.015)	0.431				
BNP	1.176 (1.064–1.299)	< 0.001	1.021 (0.877–1.188)	0.791	0.981 (0.836–1.150)	0.810
CRP	1.241 (1.104–1.395)	< 0.001	1.158 (0.988–1.358)	0.071	1.147 (0.972–1.354)	0.104
cTnT	1.077 (0.968–1.198)	0.171				
LVEF	1.010 (1.026–1.116)	0.002	1.069 (1.026–1.114)	0.002	1.071 (1.028–1.116)	0.001
Aspirin	0.450 (0.303–0.668)	< 0.001	0.602 (0.368–0.987)	0.044	0.609 (0.372–0.998)	0.049
Clopidogrel	0.402 (0.250–0.646)	< 0.001	1.202 (0.591–2.444)	0.612	1.257 (0.617–2.559)	0.529
ACEI/ARB	0.384 (0.289–0.511)	< 0.001	0.651 (0.448–0.947)	0.025	0.648 (0.448–0.936)	0.021
β-Blocker	0.382 (0.287–0.509)	< 0.001	0.805 (0.559–0.159)	0.243	0.803 (0.558–1.154)	0.236
CCB	1.825 (1.346–2.474)	< 0.001	1.437 (0.962–2.147)	0.077	1.373 (0.921–2.048)	0.120
Diabetes	1.272 (0.950–1.704)	0.106				
Hypertension	1.894 (1.369–2.621)	< 0.001	1.106 (0.709–1.724)	0.658	1.115 (0.715–1.740)	0.631
PCI	1.012 (0.989–1.016)	0.534				

Model 1^#^ used D-dimer as a continuous variable; Model 2^*^ used D-dimer as a categorical variable. *AC*CI, age-adjusted Charlson Comorbidity Index; *DBP*, diastolic blood pressure; BG, blood glucose; *BNP*, B-type natriuretic peptide; *CRP*, C-reactive protein; *LVEF*, left ventricular ejection fraction; *ACEI*, angiotensin-converting enzyme inhibitors; *ARB*, angiotensin receptor blockers; *CCB*, calcium channel blockers; *P*CI, percutaneous coronary intervention. D-dimer quartile: Q1: ≤ 0.3 μg/L; Q2: 0.31–0.46 μg/L; Q3: 0.47–0.96 μg/L; and Q4: >0.96 μg/L.

A univariate Cox regression analysis showed that the occurrence of MACEs were significantly correlated with gender [hazard ratio [HR]: 1.487, 95% CI: 1.090–2.027, *p* = 0.012], age [HR: 1.058, 95% CI: 1.046–1.070, *p* < 0.001], D-dimer [HR:1.152, 95% CI: 1.120–1.185, *p* < 0.001], fibrinogen [HR: 1.272, 95% CI: 1.154–1.402, *p* < 0.001], ACCI [HR: 1.419, 95% CI: 1.339–1.503, *p* < 0.001], BNP [HR:1.176, 95% CI: 1.064–1.299; *p* < 0.001], CRP [HR: 1.241, 95% CI: 1.104–1.395; *p* < 0.001], LVEF [HR: 1.010, 95% CI: 1.026–1.116, *p* < 0.001], aspirin [HR: 0.450, 95% CI: 0.303–0.668, *p* < 0.001], clopidogrel [HR: 0.402, 95% CI: 0.250–0.646, *p* < 0.001], angiotensin-converting enzyme inhibitor (ACEI)/angiotensin receptor blocker (ARB) [HR: 0.384, 95% CI: 0.289–0.511, *p* < 0.001], β-blocker [HR: 0.382, 95% CI: 0.287–0.509, *p* < 0.001], calcium channel blockers (CCB) [HR: 1.825, 95% CI: 1.346–2.474, *p* < 0.001], and hypertension [HR: 1.894, 95% CI: 1.369–2.621, *p* < 0.001], but not with smoking, fasting blood-glucose, aspirin, diabetes, and percutaneous coronary intervention (PCI).

A multivariate Cox regression analysis based on age, sex, ACCI, fibrinogen, CRP, LVEF, aspirin, clopidogrel, ACEI/ARB, β-blocker, hypertension, and D-dimer indicated that plasma D-dimer [HR: 1.071, 95% CI: 1.013–1.133, *p* < 0.017], ACCI [HR: 1.264, 95% CI: 1.141–1.400, *p* < 0.001], LVEF [HR: 1.069, 95% CI: 1.026–1.114, *p* = 0.001], aspirin [HR: 0.604, 95% CI: 0.369–0.990, *p* = 0.046], ACEI/ARB [HR: 0.661, 95% CI: 0.457–0.956, *p* = 0.028], and CCB [HR: 1.484, 95% CI: 1.018–2.164, *p* = 0.040] were independent risk factors for MACEs.

When using D-dimer as a categorical variable, the multivariate Cox regression analysis indicated that the hazard ratios pertaining to MACEs in patients with the Q2, Q3, and Q4 groups compared with the Q1 group were 1.348 [HR: 1.348, 95% CI: 0.649–2.799, *p* = 0.423], 1.551 [HR: 1.551, 95% CI: 0.770–3.124, *p* = 0.220], and 2.336 [HR: 2.336, 95% CI: 1.144–4.768, *p* = 0.020], respectively. Therefore, the Q4 group of patients was at the highest risk of developing MACEs, as shown in Table 4.

A univariate regression analysis assessed predictors of all-cause death (Table 5). Furthermore, Table 5 shows the final multivariate Cox proportional models of predictors for all-cause death. After adjustment for multiple relevant covariables, ACCI [HR: 1.292, 95% CI: 1.131–1.476, *p* < 0.001] and D-dimer [HR: 1.082, 95% CI: 1.021–1.147, *p* = 0.008] were still independent predictors for all-cause death in the study population. When using D-dimer as a categorical variable, the multivariate Cox regression analysis indicated that the hazard ratios pertaining to all-cause death of patients in the Q2, Q3, and Q4 groups compared with patients in the Q1 group were 1.258 [HR: 1.258, 95% CI: 0.609–2.599, *p* = 0.535], 1.463 [HR: 1.463, 95% CI: 0.726–2.947, *p* = 0.287], and 2.338 [HR: 2.338, 95% CI: 1.139–4.801, *p* = 0.021], respectively. Following the above analysis, we found that the Q4 group of patients was at the highest risk of developing all-cause death, as shown in Table 5.

**Table 5:** The univariate and multivariate Cox regression analyses of all-cause death in patients with NSTEMI.

**Variables**	**Univariable**		**Model 1** ^**#**^		Model 2*	
	**HR (95% CI)**	***P*-value**	**HR (95% CI)**	***P*-value**	**HR (95% CI)**	***P*-value**
Gender	1.692 (1.154–2.481)	0.007	0.921 (0.564–1.506)	0.744	0.920 (0.634–1.333)	0.658
Age, years	1.081 (1.065–1.098)	< 0.001	1.045 (1.017–1.073)	0.001	1.015 (0.995–1.036)	0.144
D-dimer	1.176 (1.142–1.212)	< 0.001	1.079 (1.018–1.144)	0.011	–	
Q2 vs. Q1	2.475 (0.762–8.038)	0.132	–		1.258 (0.609–2.599)	0.535
Q3 vs. Q1	7.237 (2.511–20.861)	< 0.001	–		1.463 (0.726–2.947)	0.287
Q4 vs. Q1	31.002 (11.351–84.668)	< 0.001	–		2.338 (1.139–4.801)	0.021
Smoking	0.865 (0.602–1.243)	0.434				
Fibrinogen	1.289 (1.140–1.457)	< 0.001	1.023 (0.861–1.217)	0.793	0.997 (0.874–1.139)	0.970
ACCI	1.419 (1.339–1.503)	< 0.001	1.276 (1.117–1.458)	< 0.001	1.218 (1.097–1.353)	< 0.001
BG	1.006 (0.995–1.018)	0.293				
BNP	1.134 (1.000–1.287)	0.051				
CRP	1.209 (1.037–1.395)	0.014	1.078 (0.890–1.307)	0.441	1.030 (0.849–1.248)	0.767
cTnT	1.035 (0.906–1.182)	0.614				
LVEF	0.008 (0.002–0.037)	< 0.001	0.092 (0.012–0.697)	0.021	1.074 (1.030–1.119)	0.001
Aspirin	0.308 (0.198–0.478)	< 0.001	0.522 (0.292–0.932)	0.028	0.586 (0.359–0.957)	0.033
Clopidogrel	0.315 (0.183–0.541)	< 0.001	1.066 (0.448–2.539)	0.884	1.221 (0.598–2.491)	0.584
ACEI/ARB	0.235 (0.160–0.344)	< 0.001	0.433 (0.256–0.732)	0.002	0.637 (0.440–0.922)	0.017
β-Blocker	0.285 (0.199–0.408)	< 0.001	0.764 (0.482–1.121)	0.253	0.838 (0.584–1.202)	0.337
CCB	1.732 (1.174–2.553)	0.006	1.421 (0.818–2.469)			1.351 (0.907–2.012)
Diabetes	1.035 (0.707–1.514)	0.860				
Hypertension	1.914 (1.266–2.896)	0.002	1.022 (0.565–1.849)	0.942	1.117 (0.716–1.742)	0.627
PCI	1.046 (0.972–1.025)	0.597				

Model 1^#^, using D-dimer as a continuous variable; Model 2^*^, using D-dimer as a categorical variable. *AC*CI, age-adjusted Charlson Comorbidity Index; BG, blood glucose; *BNP*, B-type natriuretic peptide; *CRP*, C-reactive protein; *DBP*, diastolic blood pressure; *LVEF*, left ventricular ejection fraction; *ACEI*, angiotensin-converting enzyme inhibitors; *ARB*, angiotensin receptor blockers; and CCB, calcium channel blockers. D-dimer quartile: Q1: ≤ 0.3 μg/L; Q2: 0.31–0.46 μg/L; Q3: 0.47–0.96 μg/L; and Q4: >0.96 μg/L.

**Figure 3:**
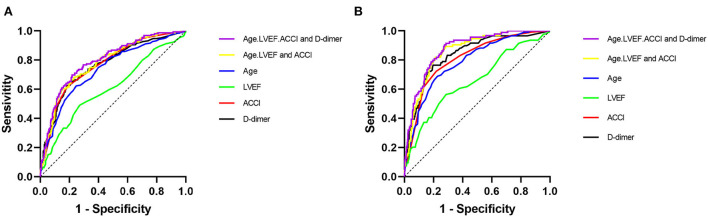
The receiver operating characteristic (ROC) curve. **(A**) MACEs, **(B)** All-cause death.

The receiver operating characteristic (ROC) curves are shown in Figure 3. An ROC analysis revealed that the area under the curve (AUC) of plasma D-dimer for MACEs [0.763 (95% CI: 0.722–0.804, *p* < 0.001)] was similar to that of ACCI [0.768 (95% CI: 0.729–0.808, *p* < 0.001)]. The AUC of plasma D-dimer was greater than that of CRP [0.580 (95% CI: 0.532–0.628, *p* = 0.001)] and LVEF [0.607 (95% CI: 0.554–0.661, *p* < 0.001)]. In addition, we used a combination of indicators to predict MACEs. We found that the AUC of the combination of D-dimer, LVEF, CRP, and ACCI [0.802 (95% CI: 0.763–0.840, *p* < 0.001)] to predict MACEs was greater than the combination of AUC of LVEF, CRP, and ACCI [0.789 (95% CI: 0.749–0.829, *p* < 0.001)] to predict MACEs.

For all-cause death, we found that the AUC of plasma D-dimer [0.836 (95% CI 0.796–0.876, *P* < 0.001)] was higher than the AUC of the ACCI [0.809 (95% CI 0.767–0.851, *P* < 0.001)], age [0.789 (95% CI 0.746–0.833, *P* < 0.001)] and LVEF [0.656 (95% CI 0.593–0.720, *P* < 0.001)]. Similarly, we used combined indicators to predict all-cause death. We found that the AUC of the combination of D-dimer, LVEF, age, and ACCI [0.875 (95% CI 0.841–0.910, *P* < 0.001)] was greater than the combination of AUC of Age, LVEF, and ACCI [0.860 (95% CI 0.824–0.896, *P* < 0.001)] to predict all-cause death.

## Discussion

Non-ST-segment elevation myocardial infarction is a common acute coronary syndrome with a high morbidity and mortality rate. The identification of methods to further improve the survival of patients remains a great challenge. In this study, we found that the ACCI and plasma D-dimer levels were independent risk factors for the occurrence of MACEs and all-cause death during long-term follow-up in Chinese patients with NSTEMI. Patients with plasma D-dimer >0.96 μg/L were at a high risk of developing MACEs and all-cause death.

The ACCI has provided a simple, readily applicable, and a valid method of estimating clinical outcomes from comorbid diseases for use in longitudinal studies (13). Our study demonstrated again that, after adjusting for age, sex, and other factors, the ACCI was still independently associated with the occurrence of MACEs and all-cause deaths. By comparing the areas under the ROC curve, D-dimer levels were superior to ACCI in predicting all-cause death. Compared with the complex calculation of ACCI based on medical history, D-dimer could be identified only by a blood test, which might be more suitable for patients with critical illnesses or speech disorders. In a real-world retrospective study, Li et al. (10) observed that patients with STEMI who had a higher plasma D-dimer might benefit from the use of thrombus aspiration during primary PCI. In addition, we used joint indicators to predict MACEs and all-cause deaths. We found that the AUC of the joint indicator was greater for MACEs and all-cause deaths. The addition of D-dimer to clinical predictors can significantly improve risk predictions for MACEs and all-cause deaths. This facilitates decision-making on treatment strategies (15).

However, there are still some disputes about the relationship between plasma D-dimer and clinical outcomes in patients with the coronary heart disease. Oldgren et al. (16) demonstrated that higher D-dimer levels are associated with worse clinical outcomes in patients with unstable coronary diseases. The greater the D-dimer level is, the greater the thrombosis load (17). Higher D-dimer levels were associated with the severity of myocardial infarction in patients with STEMI undergoing primary PCI (18). Zhao et al. (19) considered a potential benefit of lowering D-dimer levels among DM patients with *de novo* lesions. Our study included more diseases in addition to diabetes, and we used the ACCI to score common diseases. Our study reached similar conclusions in Chinese patients with NSTEMI and more strongly demonstrated the predictive value of D-dimer for clinical outcomes in patients with acute coronary syndromes.

Nevertheless, Tello-Montoliu et al. (20) did not observe any advantage of plasma D-dimer when troponin T, CRP, and NT-ProBNP were considered in patients with NSTEMI during 6 months of follow-up. This difference in studies might be due to variations in blood sampling time points and the administration of anticoagulants, such as heparin and other antithrombotic agents, used in the first 36 h, which might influence plasma D-dimer levels. In addition, a positive correlation was noted between D-dimer and other biomarkers, such as troponin T, CRP, and NT-proBNP, in patients with non-ST-elevation acute coronary syndrome (20). The GRACE score has been recognized as a powerful tool for risk assessment in patients with NSTEMI (21). Satilmisoglu et al. (22) found that D-dimer was not an independent predictor of mortality risk, but a higher D-dimer level was associated with a higher GRACE score and mortality risk in the univariate analysis. Lu et al. (23) suggested that NT-proBNP, D-dimer, and fibrinogen combined with the GRACE score exhibited a higher predictive power for the risk of MACE events in patients with NSTEMI than the GRACE score alone. The disparate conclusions of these studies might be attributed to their limited sample size and event records during the follow-up period, which precludes researchers from reaching more definitive conclusions about the predictive values of the D-dimer assay for adverse outcomes in patients with NSTEMI.

It is worth noting that only patients with a clear diagnosis of myocardial infarction were included in the present study, and those without increased troponin levels were excluded. This population selection might weaken the predictive value of troponin and BNP, so there was no overall difference in the plasma cardiac troponin-T and BNP levels among the groups in our study. In addition, we found that the patients with plasma D-dimer >0.96 μg/L had significantly higher MACEs and all-cause mortality than the other groups based on the multivariate Cox regression analysis. Similarly, Gong et al. (24) found that higher baseline D-dimer levels were related to a higher risk of MACEs in patients not taking anticoagulant drugs. In the ATLAS ACS TIMI-46 trial substudy (25), increased baseline D-dimer was associated with an increased risk of MACEs within 6 months of the ACS event, and administration of the Factor Xa inhibitor contributed to lower D-dimer levels at day 30 and 180. Therefore, we should perform a more rigorous follow-up and standardized treatment for patients at high risk (D-dimer >0.96 μg/L) to explore whether antithrombotic therapy aimed to lower D-dimer levels can improve prognosis in patients with NSTEMI.

## Limitations

There are several inherent limitations to the current study. First, this study was a single-center retrospective study with a limited sample, and patients who were lost to follow-up were excluded from this study, which may have lead to selection bias. Second, this study did not evaluate therapeutic strategies, such as optimizing medication and percutaneous coronary intervention, which have a substantial influence on clinical outcomes in patients with NSTEMI. In addition, we only observed the results of one D-dimer test after admission. It remains unclear whether monitoring and/or decreasing plasma D-dimer levels could improve clinical outcomes in patients with NSTEMI. Age can affect baseline plasma D-dimer levels (26). D-dimer levels were not age-normalized in this study. However, when age and other factors were included in the multivariate Cox regression analysis, the D-dimer level was still independently associated with MACEs and all-cause death risks.

## Conclusion

In Chinese patients with acute NSTEMI, higher D-dimer levels at admission suggest a poor long-term prognosis. Physicians should take plasma D-dimer seriously, and treatment aimed at reducing plasma D-dimer might help to improve the prognosis of Chinese patients with NSTEMI.

## Data availability statement

The original contributions presented in the study are included in the article/supplementary material, further inquiries can be directed to the corresponding author.

## Ethics statement

The studies involving human participants were reviewed and approved by Ethics Committee of Dongguan People's Hospital. The Ethics Committee waived the requirement of written informed consent for participation.

## Author contributions

XF and HW designed and performed data analysis. XF and TM wrote the manuscript. HW, SS, and BX reviewed the manuscript and supervised the work. All authors have read and approved the manuscript.

## Funding

This study was supported by research grants from the Key Projects of Guangdong provincial basic and applied basic research fund (Provincial and Municipal Joint Fund, No. 2020B1515120003).

## Conflict of interest

The authors declare that the research was conducted in the absence of any commercial or financial relationships that could be construed as a potential conflict of interest.

## Publisher's note

All claims expressed in this article are solely those of the authors and do not necessarily represent those of their affiliated organizations, or those of the publisher, the editors and the reviewers. Any product that may be evaluated in this article, or claim that may be made by its manufacturer, is not guaranteed or endorsed by the publisher.
